# Treatment of periprosthetic acetabular fractures after previous hemi- or total hip arthroplasty: Introduction of a new implant

**DOI:** 10.1007/s00064-016-0439-7

**Published:** 2016-04-01

**Authors:** H. Resch, D. Krappinger, P. Moroder, M. Blauth, J. Becker

**Affiliations:** Department of Trauma Surgery and Sports Injuries, Paracelsus Medical University, Muellner Hauptstr. 48, A-5020 Salzburg, Austria; Department of Trauma Surgery, Medical University Innsbruck, Innsbruck, Austria

**Keywords:** Mobilization, Osteoporosis, Weight-bearing, Prosthesis, Acetabulum, Mobilisierung, Osteoporose, Gewichtsbelastung, Prothese, Azetabulum

## Abstract

**Objective:**

Treatment of displaced periprosthetic acetabular fractures in elderly patients. The goal is to stabilize an acetabular fracture independent of the fracture pattern, by inserting the custom-made roof-reinforcement plate and starting early postoperative full weight-bearing mobilization.

**Indications:**

Acetabular fracture with or without previous hemi- or total hip arthroplasty.

**Contraindications:**

Non-displaced acetabular fractures.

**Surgical technique:**

Watson-Jones approach to provide accessibility to the anterior and supraacetabular part of the iliac bone. Angle-stable positioning of the roof-reinforcement plate without any fracture reduction. Cementing a polyethylene cup into the metal plate and restoring prosthetic femoral components.

**Postoperative management:**

Full weight-bearing mobilization within the first 10 days after surgery. In cases of two column fractures, partial weight-bearing is recommended.

**Results:**

Of 7 patients with periprosthetic acetabular fracture, 5 were available for follow-up at 3, 6, 6, 15, and 24 months postoperatively. No complications were recognized and all fractures showed bony consolidation. Early postoperative mobilization was started within the first 10 days. All patients except one reached their preinjury mobility level. This individual and novel implant is custom made for displaced acetabular and periprosthetic fractures in patients with osteopenic bone. It provides a hopeful benefit due to early full weight-bearing mobilization within the first 10 days after surgery.

**Limitations:**

In case of largely destroyed supraacetabular bone or two-column fractures according to Letournel additional synthesis via an anterior approach might be necessary. In these cases partial weight bearing is recommended.

## Zusammenfassung

**Operationsziel:**

Ziel ist die Behandlung von dislozierten periprothetischen Azetabulumfrakturen bei älteren Patienten. Dabei werden Frakturen des Azetabulums unabhängig vom Frakturmuster durch Einsetzen einer sonderangefertigten Azetabulumabstützpfanne stabilisiert und eine frühe postoperative Mobilisation unter Vollbelastung begonnen.

**Indikationen:**

Azetabulumfraktur mit oder ohne vorherige Hüftendoprothetik.

**Kontraindikationen:**

Nichtdislozierte Azetabulumfrakturen.

**Operationstechnik:**

Zugang nach Watson-Jones, um die Erreichbarkeit des vorderen und supraazetabulären Anteils des Darmbeins zu ermöglichen. Winkelstabile Positionierung der Azetabulumabstützpfanne ohne Frakturreposition. Zementieren einer Polyethylenpfanne in die Metallplatte und Reposition der femoralen Prothesenkomponenten.

**Weiterbehandlung:**

Mobilisation unter Vollbelastung innerhalb der ersten 10 Tage nach Operation. In Fällen einer 2‑Pfeiler-Fraktur mit ungenügender Schraubenzahl im stabilen Knochen wird eine Teilbelastung empfohlen.

**Ergebnisse:**

Von insgesamt 7 Fällen mit periprothetischer Azetabulumfraktur konnten 5 Patienten 3, 6, 6, 15 und 24 Monate postoperativ nachuntersucht werden. Es gab keine nennenswerten Komplikationen. Alle Frakturen zeigten eine knöcherne Konsolidierung. Eine frühe postoperative Mobilisation wurde in den ersten 10 Tagen begonnen und alle Patienten außer einem erreichten ihren ursprünglichen Mobilitätsgrad.

**Schlussfolgerung:**

Dieses individuelle, neuartige Implantat ist für dislozierte Azetabulumfrakturen und periprothetische Frakturen bei Patienten mit osteoporotischem Knochen entwickelt worden. Es verspricht hoffnungsvollen Benefit aufgrund der frühen Vollmobilisation innerhalb der ersten 10 Tage nach Operation.

**Einschränkungen:**

Bei stark zerstörten supraazetabulären Knochen- oder 2-Pfeiler-Frakturen nach Letournel könnte eine zusätzliche Synthese über einen anterioren Zugang notwendig sein. In diesen Fällen wird eine Teilbelastung empfohlen.

## Introductory remarks

Periprosthetic acetabular fractures are severe complications of hemi- (HA) or total hip arthroplasty (THA), and are on the rise in terms of occurrence and recognition [1–5]. As the function of implants in hip replacement is based on the bone–cement or bone–prosthesis fixation, a fracture that interrupts this fixation presents a challenging situation. In the presence of osteoporosis, even a fall from a standing position can lead to comminuted acetabular fractures with poor prognosis [6–10]. Different management approaches for stabilization of the acetabular component using dual plates and cages have been described in the literature. In the case of a structural bone defect, allograft treatment has been attempted [11–16].

**Fig. 1:**
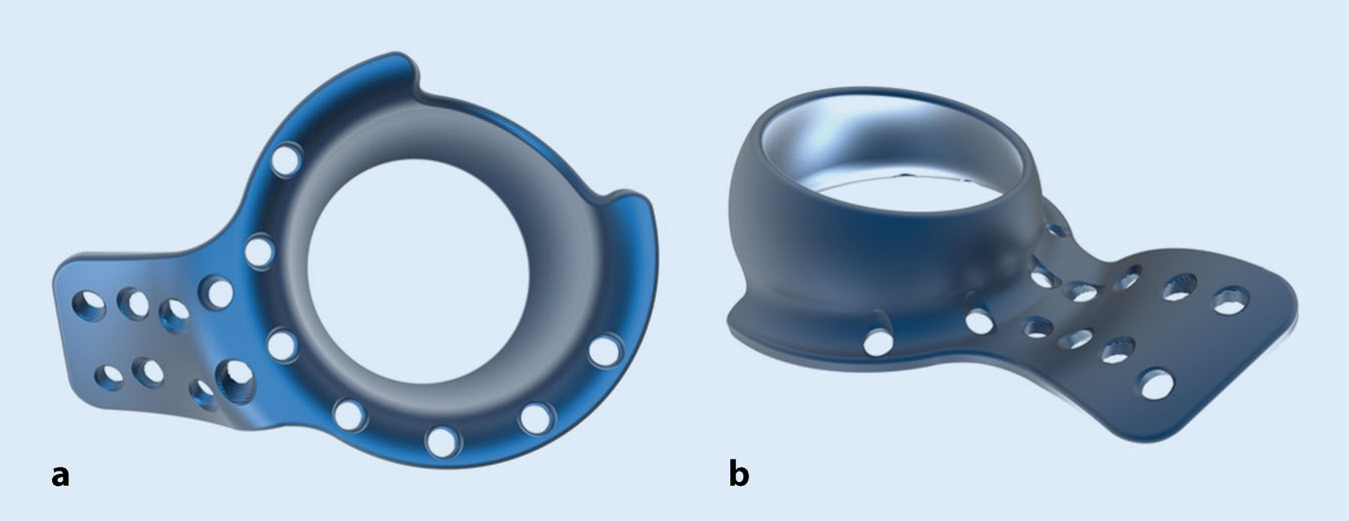
Custom-made roof-reinforcement plate showing the outer **(a)** and inner **(b)** surface with angle-stable screw holes. Courtesy of 41medical AG, Bettlach, Switzerland

Nevertheless, the results of revision surgery in HA or THA with acetabular discontinuity are poor, and conservative treatment may not be an adequate alternative [17–20]. Lower limb extension may seem to be an option, but in terms of limited physiologic tolerance in elderly patients, such treatment depicts a considerable health risk due to prolonged immobilization [21, 22]. Therefore, acetabular implants favoring stable fixation and immediate postoperative mobilization with full weight-bearing are thought to be the solution. For this purpose, a custom-built roof-reinforcement plate was designed in an attempt to provide sufficiently stable fixation at the intact iliac bone, in order to allow for early postoperative full weight-bearing in periprosthetic acetabular fractures (Fig. 1a, b). The purpose of this article is to provide a description of the novel implant and describe the surgical technique.

## The custom-built roof-reinforcement implant

The designed plate by itself has an outer diameter of 50 mm and an inner diameter of 48 mm, which perfectly fits cemented cups of 46 mm. On the cranial side, the cage is extended by a fin to provide sufficient fixation at the intact iliac bone by means of eight angle-stable 3.5-mm screws aimed in different directions. The inner ring is outfitted with another seven holes for 3.5-mm angle-stable screws to provide stabilization for the anterior and posterior column, as well as the acetabular roof. As reaming of the fractured acetabulum is performed up to 52 mm, only one size is necessary for all cases. According to preoperative planning based on CT scans, left and right implants are needed due to the fin of the cage (Fig. 1a, b).

## Surgical principle and objective

Treatment of displaced acetabular fractures with or without previous hip replacement in elderly patients. The custom-made acetabulum roof-reinforcement plate maintains stable acetabular fixation and allows immediate postoperative mobilization at least in most cases. The implant can be used in periprosthetic acetabular fractures, as well as in the presence of isolated displaced acetabular fractures requiring surgical stabilization and hip arthroplasty.

## Advantages

- Compared to the transgluteal approach (Bauer) the classic anterolateral approach (Watson-Jones) is used to provide better access to the anterior and middle supraacetabular part of the iliac bone
- One-stage procedure
- In cases with isolated displaced acetabular fractures, the femoral head can be used as autograft after resection in the presence of bone defects
- No donor site morbidity
- Limited surgery time and limited blood loss
- Immediate postoperative mobilization

## Limitations

- In case of largely destroyed supraacetabular bone or in case of a two column fracture according to Letournel [23] additional osteosynthesis might be necessary. In these cases partial weight bearing is recommended

## Indications

- Displaced acetabular fractures without previous hip replacement
- Periprosthetic acetabular fracture in HA
- Periprosthetic acetabular fracture in THA
- Central pelvic dislocation of the femoral head and acetabular protrusion after HA
- Age of 65 years or older, depending on bone quality
- Pretraumatic mobility dependent on a walker at the most
- Non-union of acetabulum fractures after open reduction internal fixation (ORIF)

## Contraindications

- Poor general health situation
- Active or latent infection
- Sepsis
- Allergy against implant material
- Local bone tumors or cysts
- Age below 65 years
- Non-displaced acetabular fractures

## Patient information

- Possible delayed or absent healing of osteoporotic bone
- Possible intolerance to the implant
- Possible wound healing disturbances, sensibility disturbances, and/or circulation disorders with need for surgical revision
- General risks of surgery
- Longer surgical time due to cage fixation

## Preoperative workup

- Clinical assessment of pelvic stability
- Documentation of the patient’s preinjury mobility status
- X-ray of the pelvis and hip with AP and oblique views
- CT scans of the involved hip in three planes for implant planning
- Documentation of the sensibility and circulation of the foot
- General preparations for surgery

## Instruments and implants

- Basic set of surgical instruments for pelvic surgery
- Patient-fitted roof-reinforcement plate 3.5 based on preoperative CT scans
- Screwdriver hex 2.5 mm with screwdriver bit and helve
- Screwdriver star drive T15 with screwdriver bit
- Torque limiter 1.5 Nm
- 3.5-mm hex self-cutting angle-stable screws (L = 10–95 mm)
- 3.5 mm star drive self-cutting angle-stable screws (L = 10–95 mm)

## Anesthesia and positioning

- Endotracheal intubation or larynx mask anesthesia
- Perioperative single shot of antibiotic (e. g., 2 g cefacolin i. v.)
- Supine position
- The hip, iliac crest, and proximal part of the femur are disinfected at once

## Surgical technique

**Fig. 2:**
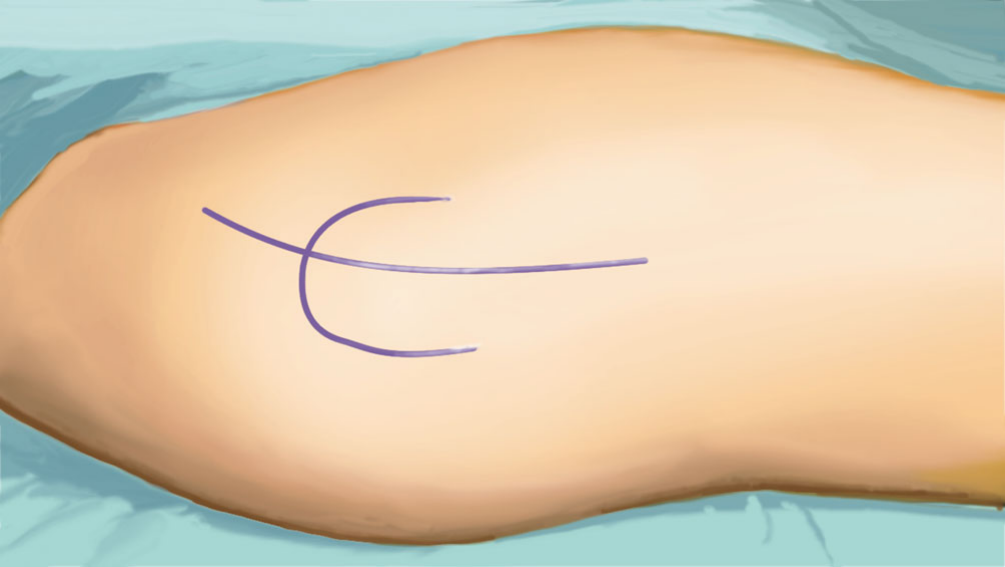
The surgical intervention takes place in general anesthesia and supine position. As surgical approach serves the classic anterolateral Watson-Jones approach which provides a perfect accessibility to the anterior and middle supra-acetabular part of the iliac bone. The landmarks for the skin incision include the anterior superior iliac spine, the greater trochanter and the plain of the femur. The incision starts 2.5 cm posterior and inferior to the anterior superior iliac spine and is slightly curved dorsally to the greater trochanter prolonged to the femoral shaft for about 5 cm

**Fig. 3:**
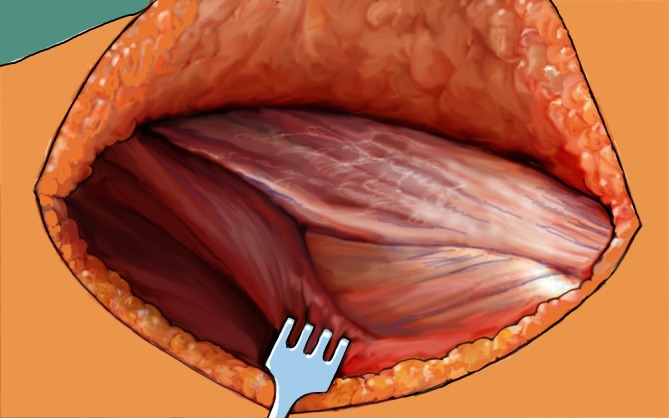
The triangle of the tensor fascie latae, gluteus medius, and lateral vastus muscle is then identified and opened midway between the anterior spine and greater trochanter. Subsequently, the ridge of the lateral vastus muscle is revealed

**Fig. 4:**
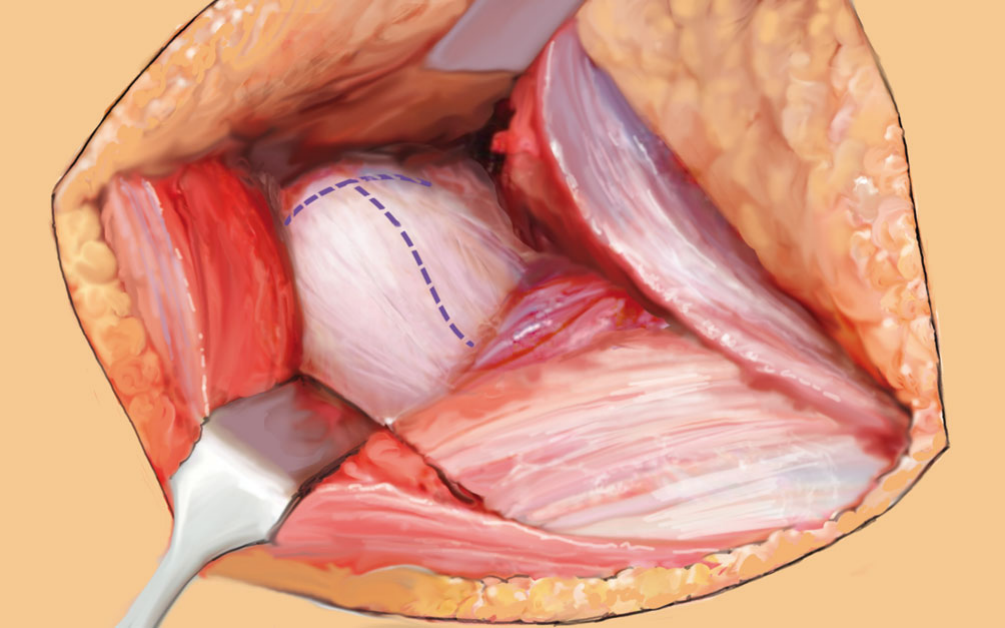
After exposure of the prosthesis, the leg is brought into second position while dislocating carefully the prosthetic head. Retractors are placed anteriorly, posteriorly, and inferiorly, to optimize visualization of the acetabular fracture. In patients with non-periprosthetic fractures, the capsule is exposed and resected by a T-shape incision. Furthermore, femoral neck osteotomy and acetabular cartilage removal is performed before stepwise socket reaming, starting from 44 up to 52 mm, and implant insertion. In periprosthetic fractures after total hip arthroplasty, the acetabular component is removed with or without all the cement, depending on the type of prosthesis. In case of hemiarthroplasty, only the prosthetic head has to be removed

**Fig. 5:**
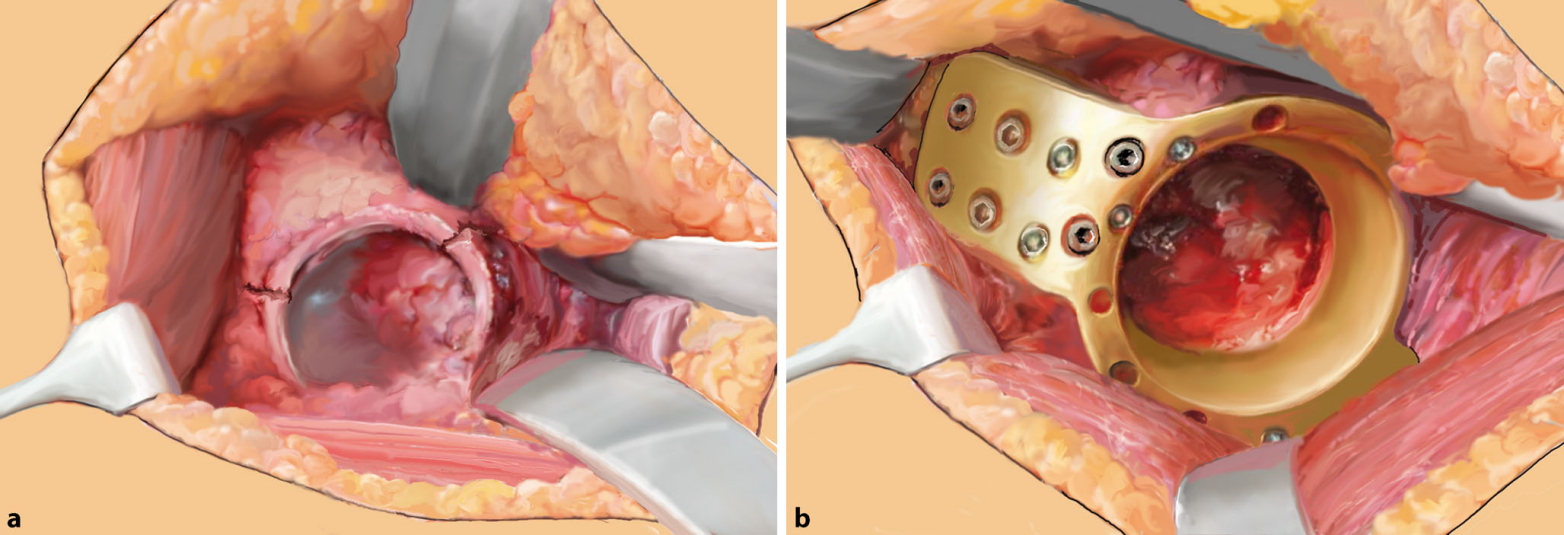
Next, 5 cm of the anterosuperior and superior part of the acetabular roof are freed from soft-tissue for positioning of the fin. Regardless of fracture type, the roof-reinforced plate is installed without any anterior-superior reduction of the fracture and carefully pressed with a tappet to the acetabular roof for good contact. In case of an anterior column fracture reaching up superiorly into the acetabular roof, the fin is positioned further posteriorly to purchase screw fixation. The fin is then fixed to the iliac bone by inserting 3.5-mm angle-stable screws aimed in different directions. The drill is guided by the 3.5-mm boring bush and should always penetrate the opposite cortex. The length of the screws is determined by means of a measuring instrument. Furthermore, additional screws are inserted through the upper holes in the ring and, if possible, through inferior holes as well

**Fig. 6:**
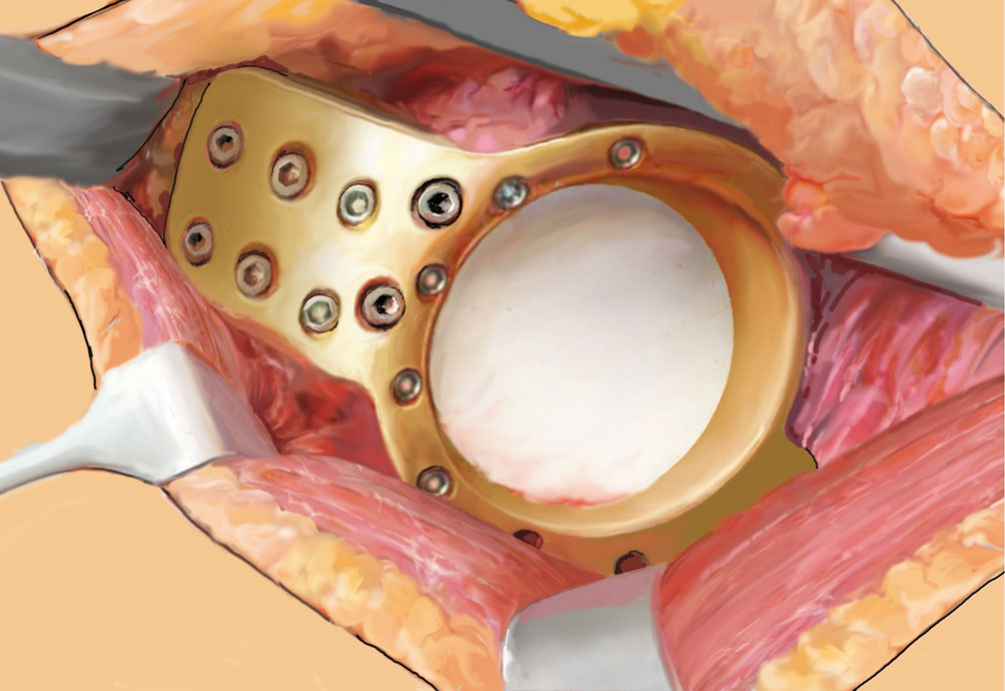
In periprosthetic fractures no bon grafting is performed. A Prolene® mesh graft (Ethicon, Johnson & Johnson Medical, Norderstedt, Germany) is now sutured to the inner aperture of the implant ring to cover it and prevent cement leakage into the pelvis. In cases of an isolated acetabular fracture, slices of the resected femoral head are placed at the bottom of the implant ring to provide better bony stabilization and improve bony healing

**Fig. 7:**
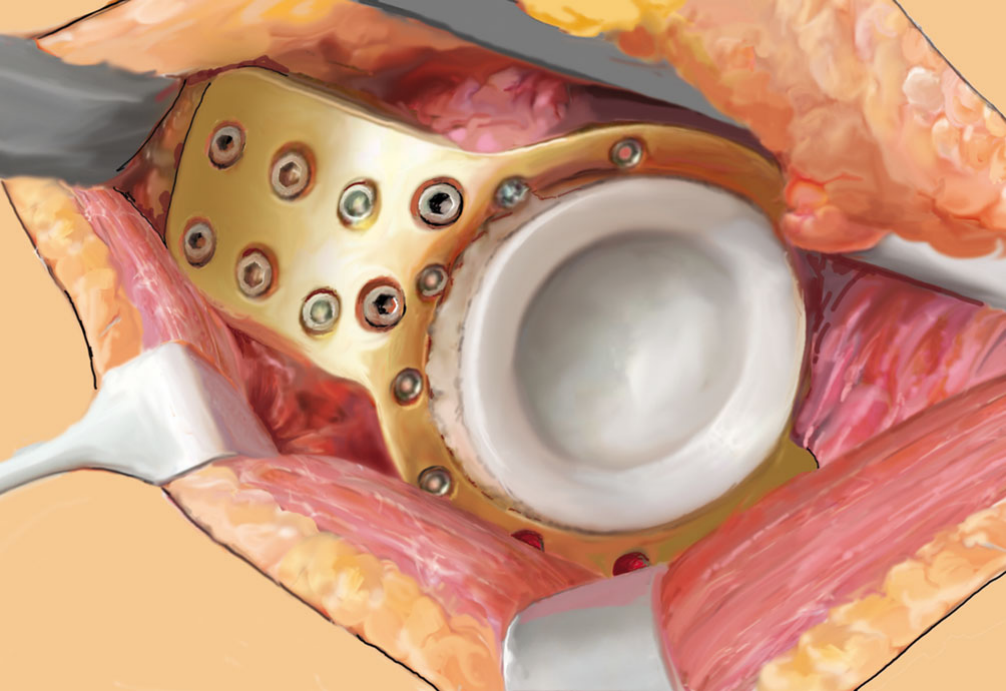
Subsequently, a polyethylene cup with diameter 46 mm is cemented into the metal cage and the prosthetic femoral components are restored in typical manner

(Fig. 2, 3, 4, 5, 6, 7)

## Postoperative management

- Passive and active motion should be exercised up to the onset of pain and intensified step-by-step directly after surgery.
- Early mobilization with full weight-bearing is started within the first few days with use of a walking aid (crutches, walking frame, cane).
- In cases of destruction of the supraacetabular bone or with two column fractures, postoperative CT scan is recommended in order to check the number of screws positioned in stable bone. Postoperative mobilization depends on this information (full or partial weight-bearing).

## Errors, hazards, and complications

- If the inserted Prolene® mesh graft leaks, a cement outflow into the pelvis is possible.
- As joint stability depends on the positioning of the polyethylene cup, increased attention has to be paid during cementing.
- In the case of poor positioning of the cemented cup, dislocation of the prosthetic head can occur.
- Deep wound infection should be treated by early surgical debridement and appropriate antibiotic treatment.
- Incomplete healing of the bone in situations of osteoporosis, partial weight-bearing is recommended.
- Surgical repetition is possible. However, after failure of initial surgery, careful reassessment of possible causes of failure is mandatory.
- In cases with a fractured acetabular roof, a postoperative CT scan should be performed in order to check screw fixation and stability. Postoperative mobilization with full or partial weight-bearing depends on this information.

## Results

**Fig. 8:**
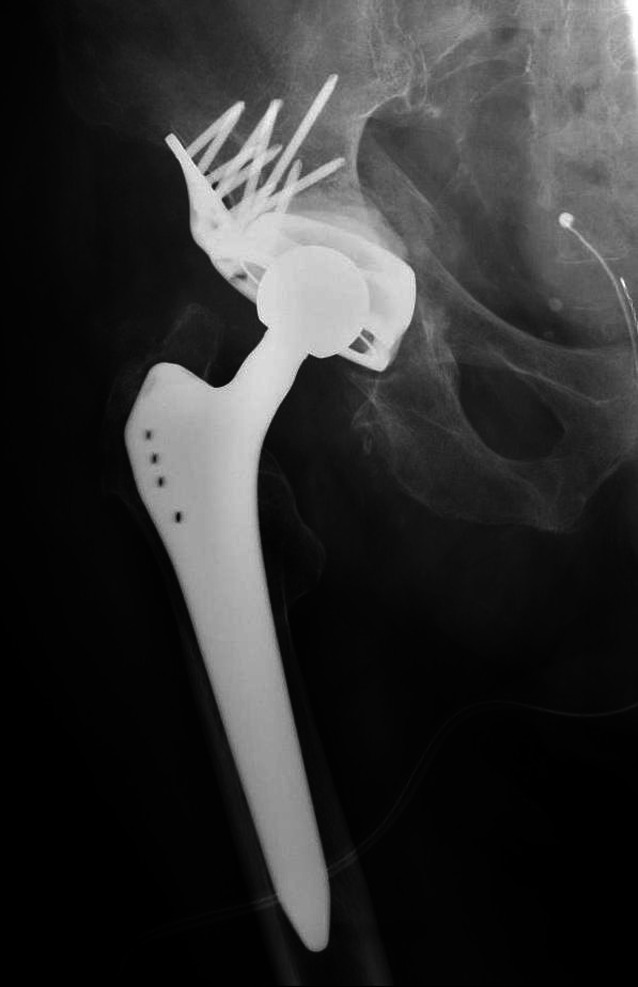
Postoperative AP X‑ray

Between 2010 and 2014, 7 patients suffering from a periprosthetic fracture were treated using the roof-reinforcement plate. At the time of surgery, the average age of these 7 patients was 80 years (range 65–91 years). Previously, 5 patients had undergone HA and 2 THA. All except one patient with HA had a transverse fracture; the one exception had a T-fracture. Of the two patients with THA, one showed an anterior column fracture in combination with a fracture of the quadrilateral plane; the other patient had a central dislocation of the acetabular components without fracture of the two columns. Postoperatively, all patients were allowed for full weight-bearing. Only 5 patients were available for follow-up (FU), two had died in the meantime. FU of the single patients was performed after 3, 6, 6, 15, and 24 months postoperatively. At this time, X‑rays in 4 patients and a CT scan in one were available. In all patients, bony consolidation could be proven, without any signs of loosening (Fig. 8). All patients except one were able to reach their preinjury mobility level. The patient who did not reach the former mobility level had to use a cane, which he did not need before surgery. Of the remaining 4 patients, 2 used a cane, one a walking frame, and one did not use a walking aid at all.

## Discussion

Compared to the increasing number of acetabular fractures in the elderly, patients with periprosthetic acetabular fractures are still not very common. Considering a time period of almost 4 years in two level I trauma centers, the authors can report only on 7 patients. Due to the advanced age of the patients, with an average age of 80 years, only 5 were available for FU. Two patients had died in the meantime. In a group of patients with such advanced age it is sometimes difficult to follow-up for a long time period. The authors are aware that this is a limitation of this paper.

In the literature it is reported that the typical acetabulum fracture in osteoporotic bone conditions involves the anterior column associated with a fracture of the quadrilateral plane [5]. This seems to be different with periprosthetic fractures. Only one of the 7 patients showed the described fracture type, whereas among the other 6, a transverse fracture was found in 5 patients and a T-fracture in one. Concerns are reported in the literature regarding stable fixation of the acetabular component, recommending additional cables or plates [5, 10]. The design of the described roof-reinforcement plate is such that all the stability is provided by fixation of the fin of the cage at the intact iliac bone by eight multidirectional angle-stable screws. Additional stability is provided by up to seven angle-stable screws through upper, anterior, and posterior holes of the ring. The stability of fixation allows immediate postoperative full weight-bearing, at least in most cases. Fixation of a fractured anterior column can be performed by anterior screws, but this does not enhance primary stability of the cage. The results of a series of 30 consecutive patients of the same age (average 79 years) suffering from acetabulum fractures without previous prostheses have shown that the stability provided by the fixation described above was sufficient for early full weight-bearing (paper under review). No loosening signs were found in any case. New and modern titanium fixators with multidirectional interlocking screws inserted by a minimally invasive procedure might be an alternative [24]; however, in the authors’ experience, in periprosthetic fractures the quadrilateral plane is commonly destroyed and associated with a dome fragment of the acetabulum. Furthermore, due to advanced head protrusion in the case of HA, the bone of the quadrilateral plane is thin and of very poor quality, rendering stable fixation even with the new plates difficult.

## Conclusion

In summary, this report demonstrates that this custom-built roof-reinforcement plate is a beneficial addition to the treatment spectrum for elderly patients with previous hip replacement, especially for patients with periprosthetic acetabular discontinuity after THA and HA. Early mobilization with full weight-bearing within the first 10 days after surgery can be achieved, at least in most cases. However, short- and long-term results from higher numbers of cases are needed in order to draw conclusions on the mechanical behavior of this custom-made reconstructive implant over time.

## References

[1] McElfresh EC, Coventry MB (1974). Femoral and pelvic fractures after total hip arthroplasty. J Bone Joint Surg Am.

[2] Miller AJ (1972). Late fracture of the acetabulum after total hip replacement. J Bone Joint Surg Br.

[3] Silvello L, Scarponi R, Lucia G, Guazzetti R (1985). Traumatic loosening of a prosthetic acetabular cup in a young patient. Ital J Orthop Traumatol.

[4] Ochs BG, Marintschev I, Hoyer H, Rolauffs B, Culemann U, Pohlemann T (2010). Changes in the treatment of acetabular fractures over 15 years: Analysis of 1266 cases treated by the German Pelvic Multicentre Study Group (DAO/DGU). Injury.

[5] Mears DC (1999). Surgical treatment of acetabular fractures in elderly patients with osteoporotic bone. J Am Acad Orthop Surg.

[6] Desai G, Ries MD (2011). Early postoperative acetabular discontinuity after total hip arthroplasty. J Arthroplasty.

[7] Gelalis ID, Politis AN, Arnaoutoglou CM, Georgakopoulos N, Mitsiou D, Xenakis TA (2010). Traumatic periprosthetic acetabular fracture treated by acute one-stage revision arthroplasty. A case report and review of the literature. Injury.

[8] Gras F, Marintschev I, Klos K, Fujak A, Muckley T, Hofmann GO (2010). Navigated percutaneous screw fixation of a periprosthetic acetabular fracture. J Arthroplasty.

[9] Laflamme GY, Belzile EL, Fernandes JC, Vendittoli PA, Hebert-Davies J (2015). Periprosthetic fractures of the acetabulum during cup insertion: posterior column stability is crucial. J Arthroplasty.

[10] Mears DC, Velyvis JH (2002). Acute total hip arthroplasty for selected displaced acetabular fractures: two to twelve-year results. J Bone Joint Surg Am.

[11] Helfet DL, Ali A (2004). Periprosthetic fractures of the acetabulum. Instr Course Lect.

[12] Jeffery M, Scott G, Freeman M (2003). Failure of an uncemented non-porous metal-backed prosthesis with augmentation using impacted allograft for acetabular revision 12- to 17-year results. J Bone Joint Surg Br.

[13] Petrera P, Rubash HE (1995). Revision Total Hip Arthroplasty: The Acetabular Component.. J Am Acad Orthop Surg.

[14] DeBoer DK, Christie MJ, Brinson MF, Morrison JC (2007). Revision total hip arthroplasty for pelvic discontinuity. J Bone Joint Surg Am.

[15] Berry DJ (2004). Antiprotrusio cages for acetabular revision. Clin Orthop Relat Res.

[16] Ochs BG, Schmid U, Rieth J, Ateschrang A, Weise K, Ochs U (2008). Acetabular bone reconstruction in revision arthroplasty: a comparison of freeze-dried, irradiated and chemically-treated allograft vitalised with autologous marrow versus frozen non-irradiated allograft. J Bone Joint Surg Br.

[17] Chatoo M, Parfitt J, Pearse MF (1998). Periprosthetic acetabular fracture associated with extensive osteolysis. J Arthroplasty.

[18] Masri BA, Meek RM, Duncan CP (2004). Periprosthetic fractures evaluation and treatment. Clin Orthop Relat Res.

[19] Old AB, McGrory BJ, Peterson CA, Lebar RD, Goudreau FS (2008). Locked inferior fracture dislocation after total hip arthroplasty. J Arthroplasty.

[20] Sanchez-Sotelo J, McGrory BJ, Berry DJ (2000). Acute periprosthetic fracture of the acetabulum associated with osteolytic pelvic lesions: a report of 3 cases. J Arthroplasty.

[21] Matta JM, Mehne DK, Roffi R (1986). Fractures of the acetabulum. Early results of a prospective study. Clin Orthop Relat Res.

[22] Spencer RF (1989). Acetabular fractures in older patients. J Bone Joint Surg Br.

[23] Letournel E (1980). Acetabulum fractures: classification and management. Clin Orthop Relat Res.

[24] Culemann U, Holstein JH, Kohler D, Tzioupis CC, Pizanis A, Tosounidis G (2010). Different stabilisation techniques for typical acetabular fractures in the elderly--a biomechanical assessment. Injury.

